# Index-Catalogue of the Library of the Surgeon-General’s Office, United States Army

**Published:** 1890-01

**Authors:** 


**Index-Catalogue of the Library of the Surgeon–General's Office, United States Army.** Compiled by JOHN S. BILLINGS, Surgeon, U. S. A. Authors and Subjects. Vol. X. Pp. 1,059. O.—Pfutsch. Washington: Government Printing Office. 1889.

This volume includes 7,658 author-titles, representing 2,905 volumes and 7,282 pamphlets. It also includes 14,265 subject-titles of separate books and pamphlets, and 29,421 titles of articles in periodicals. The publication of this work was begun in 1880; ten volumes have been issued—an average of one a year. The total number of titles to date is given in a table in the letter of the bibliographer transmitting the work to the Surgeon-General, from which it is ascertained that there have been published already, under the head of author-titles, 107,788 titles, representing 54,298 volumes and 93,002 pamphlets; under the head of subject-titles, 107,419 book-titles and 336,772 journal articles, besides 4,335 portraits. From this may be gained an idea of the stupendous character of the work necessary to prepare this invaluable index of medical literature, and it reflects the greatest credit upon its author, to whom the country properly accredits the distinction of the greatest bibliographer in the world.

